# Monitoring of Vegetation Disturbance and Restoration at the Dumping Sites of the Baorixile Open-Pit Mine Based on the LandTrendr Algorithm

**DOI:** 10.3390/ijerph19159066

**Published:** 2022-07-25

**Authors:** Junting Guo, Quansheng Li, Huizhen Xie, Jun Li, Linwei Qiao, Chengye Zhang, Guozhu Yang, Fei Wang

**Affiliations:** 1 State Key Laboratory of Water Resource Protection and Utilization in Coal Mining, Beijing 102209, China; junting.guo.a@chnenergy.com.cn (J.G.); quansheng.li@chnenergy.com.cn (Q.L.); 20047006@chnenergy.com.cn (F.W.); 2 National Institute of Low Carbon and Clean Energy, Beijing 102211, China; 3 College of Geoscience and Surveying Engineering, China University of Mining and Technology, Beijing 100083, China; huizhenxc@student.cumtb.edu.cn (H.X.); zqt1900204087g@student.cumtb.edu.cn (L.Q.); czhang@cumtb.edu.cn (C.Z.); 4 State Grid General Aviation Co., Ltd., Beijing 102209, China; gzyang3912@163.com

**Keywords:** LandTrendr, vegetation restoration, open-pit mine, dumping site

## Abstract

Overstocked dumping sites associated with open-pit coal mining occupy original vegetation areas and cause damage to the environment. The monitoring of vegetation disturbance and restoration at dumping sites is important for the accurate planning of ecological restoration in mining areas. This paper aimed to monitor and assess vegetation disturbance and restoration in the dumping sites of the Baorixile open-pit mine using the LandTrendr algorithm and remote sensing images. Firstly, based on the temporal datasets of Landsat from 1990 to 2021, the boundaries of the dumping sites in the Baorixile open-pit mine in Hulunbuir city were extracted. Secondly, the LandTrendr algorithm was used to identify the initial time and duration of vegetation disturbance and restoration, while the Normalized Difference Vegetation Index (NDVI) was used as the input parameter for the LandTrendr algorithm. Thirdly, the vegetation restoration effect at the dumping sites was monitored and analyzed from both temporal and spatial perspectives. The results showed that the dumping sites of the Baorixile open-pit mine were disturbed sharply by the mining activities. The North dumping site, the South dumping site, and the East dumping site (hereinafter referred to as the North site, the South site, and the East site) were established in 1999, 2006, and 2010, respectively. The restored areas were mainly concentrated in the South site, the East site, and the northwest of the North site. The average restoration intensity in the North site, South site, and East site was 0.515, 0.489, and 0.451, respectively, and the average disturbance intensity was 0.371, 0.398, and 0.320, respectively. The average restoration intensity in the three dumping sites was greater than the average disturbance intensity. This study demonstrates that the combination of temporal remote sensing images and the LandTrendr algorithm can follow the vegetation restoration process of an open-pit mine clearly and can be used to monitor the progress and quality of ecological restoration projects such as vegetation restoration in mining areas. It provides important data and support for accurate ecological restoration in mining areas.

## 1. Introduction

In the process of open-pit coal mining, surface stripping and land occupation cause direct damage to the vegetation coverage in the mining areas and seriously affect the quality of the local environment [1]. Dumping sites as a form of land occupation are key areas of ecological restoration associated with the open-pit coal mines. The monitoring of vegetation disturbance and restoration at the dumping sites of open-pit coal mines has important guiding significance for the accurate planning of ecological restoration in mining areas and the quality improvement of the local environment.

The growth of vegetation in a mining area is comprehensively determined by natural conditions such as climate, soil, and hydrology. Therefore, vegetation is often used as the monitoring object to determine the effect of ecological restoration in a mining area [2,3]. It is important to note, in particular, that although soil is one of the main factors affecting NDVI and spectral information captured using satellites, its effect is slow in temporal series. Comparatively, the effect of mining disturbance and restoration on NDVI is strong in temporal series. Because these two effects are different in magnitude, this paper does not focus on the effect of the soil on NDVI. Many scholars have investigated the temporal and spatial variation characteristics of vegetation in mining areas.

The Normalized Difference Vegetation Index (NDVI) is a comprehensive measure of leaf density and greenness of a vegetation canopy, which can well characterize vegetation growth [4]. It is thus an effective index to monitor the growth of vegetation in an ecological environment [5,6,7]. Landsat remotely sensed images can be used to obtain the NDVI in temporal series and analyze vegetation changes objectively and directly [8,9,10]. At present, most scholars use the NDVI as an index to analyze vegetation changes. For instance, NDVI, mixed pixel decomposition, change trend calculation, and transition matrices were used to explore the change and stability of vegetation coverage after land reclamation at the Antaibao Open-pit mine, China [11].The NDVI was used to evaluate and analyze the vegetation coverage of the Velká Podkrušnohorská dump, a brown coal dumping site, over 25 years, from 1984 to 2009, based on Landsat images [12].The NDVI was also used to monitor the Athabasca oil sands in Canada and analyze the factors causing vegetation disturbances, and it was found that human activities and climate change were the major responsible [13]. By selecting the characteristic threshold, the characteristics of land damage and the reclamation process at pixel scale were analyzed based on NDVI temporal series, and the disturbed area and interannual information about the disturbance were extracted [14]. Landsat images were used to identify disturbances caused by mining in the Appalachian mining area in the United States. It was found that the NDVI and other remote sensing indices can identify mining disturbances, and the object-oriented classification method can improve the recognition accuracy of mining disturbances [15]. The NDVI and other vegetation indices were also used to study the Seyitomer coal mine in Turkey based on Landsat data, and the analysis showed a general increasing trend of the vegetation coverage in the reclaimed land [16]. Eight vegetation indices, including the normalized vegetation index (NDVI) and other vegetation indices, were used to compare 100 reclaimed forest types in a north open-pit mine in Bohemia, Czech Republic, and evaluate the restoration of vegetation in 10 years [17]. The pixel dichotomy model was used to study the vegetation quality in semi-arid areas by using the NDVI, and the correctness of the method was verified by using the measured data [18]. The change of vegetation coverage was monitored by calculating the broadband and narrowband NDVI of sparse vegetation in the mining area [19].

Some other scholars used vegetation coverage (FVC), the copper stress vegetation index (CSVI), and other remote sensing methods to analyze vegetation changes [20]. The methods of contribution quantification and significance test were used to identify the spatial range of mining disturbance based on FVC [21]. The CSVI was constructed to characterize the degree of vegetation stress caused by copper [22]. Landsat images t different phases were used to classify the vegetation damage in a mining area and evaluate the vegetation change and damage [23]. Some remote sensing methods were used to study vegetation restoration of a mine dump in East Germany [24]. The analysis of the trend of vegetation change in eastern Inner Mongolia showed that the vegetation coverage in the mining area decreased, and the correlation between vegetation change and precipitation in the mining area was obvious [25]. The spatial distribution of vegetation coverage in the Pingshuo open-pit coal mine area was analyzed based on remote sensing images from 1992 to 2015. The results showed that the reclamation project greatly increased the vegetation coverage in the coal mine area, and the vegetation showed a favorable development trend [26].

To sum up, so far, investigations mainly used vegetation indices to analyze the temporal and spatial variation characteristics of vegetation growth in mining areas. The NDVI can reflect the comprehensive status of vegetation and has been widely used to monitor the vegetation in mining areas [27,28,29], as well as in other areas [30,31,32]. Moreover, investigations were mainly conducted on whole mining areas, whereas research regarding dumping sites is scarce. However, in the vegetation restoration of mining areas, dumping sites are the main targets of restoration.

In recent years, the LandTrendr algorithm has been proven to be useful to detect changes of the vegetation index in time series [33]. It is included online in the Google Earth Engine (GEE) [34] (Code link of LandTrendr algorithm: https://code.earthengine.google.com/f03606979139787c0c18f4efeeed76d6 (accessed on 2 April 2022)). The algorithm can monitor the vegetation changes at a dumping site in the process of ecological restoration. It has the advantage of allowing online calculation and is therefore an important tool for the monitoring of vegetation disturbance and restoration in mining areas.

This study focused on the dumping sites of the Baorixile open-pit mine. Based on the temporal datasets of Landsat from 1990 to 2021, the boundaries of the dumping sites were extracted year by year, and their temporal and spatial variation characteristics were analyzed. The LandTrendr algorithm was used to analyze the change patterns of vegetation at each dumping site on the basis of the NDVI. The vegetation disturbance and restoration of the dumping sites were monitored and analyzed from the temporal and spatial perspectives.

## 2. Study Area and Datasets

### 2.1. Study Area

**Figure 1:**
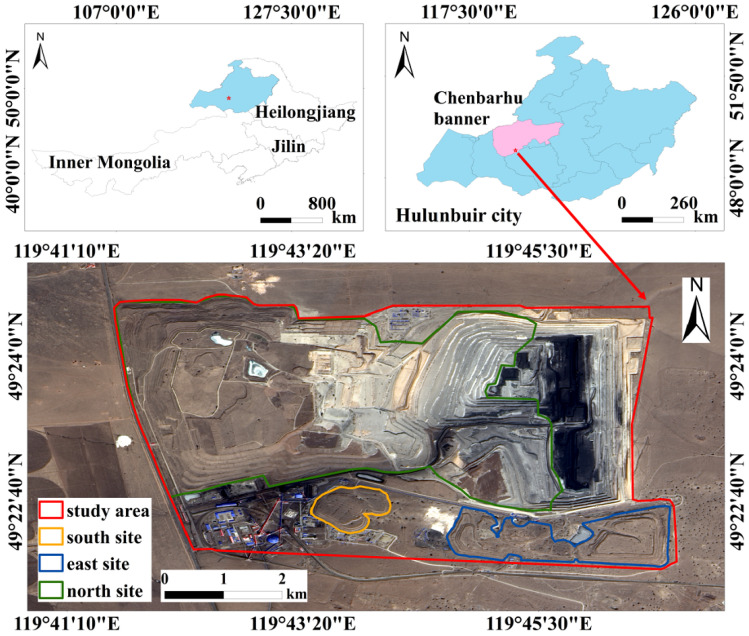
Location of the study area.

The Baorixile open-pit mine is located in Chenbalhu Banner, Hulunbuir, Inner Mongolia, China. The climate is a temperate continental monsoon type, and the annual average temperature is about −2.6 °C. The total annual precipitation usually ranges from 290 mm to 350 mm. The terrain is mainly hilly, with an average altitude ranging from 600 m to 650 m. The vegetation type in the mining area is mainly meadow grassland. The construction of the Baorixile open-pit mine was officially started in September 1998, transferred to the trial production stage in October 2000, and officially completed in April 2001. This mining area has one open-pit stope and three dumping sites, namely, the North site, the South site, and the East site. The location of the study area is shown in Figure 1.

### 2.2. Datasets

The remotely sensed images used in this study were acquired by Landsat 5, Landsat 7, and Landsat 8 and were downloaded and computed from the GEE. In this study, Landsat data were mainly used to extract the spatial ranges of the dumping sites in the mining area and retrieve the NDVI. The Landsat 5 and Landsat 7 images were used from 1990 to 2012, while the Landsat 8 images were used from 2013 to 2021. Considering the growth cycle of the vegetation, the selected image time was from June to August every year. The band information of the images used for calculating the NDVI in the study is shown in Table 1.

## 3. Methods

**Figure 2:**
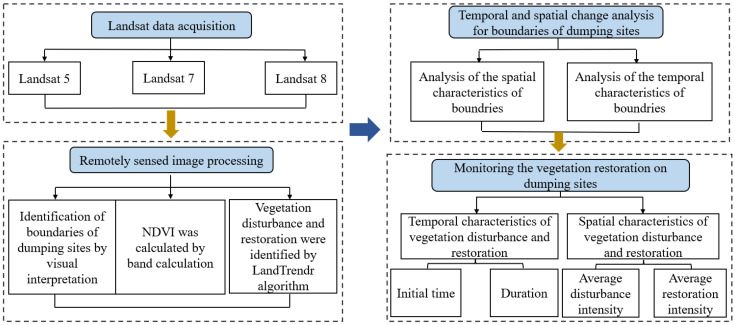
Process of monitoring and assessing the vegetation disturbance and restoration.

The technical procedure of this study mainly included four steps: (1) Landsat data acquisition; (2) Remotely sensed image processing; (3) Temporal and spatial change analysis for boundaries of the dumping sites; (4) Monitoring the vegetation restoration at the dumping sites. The technical route is shown in Figure 2.

### 3.1. Extraction of the Boundaiesy of the Dumping Sites

There are obvious differences between the ground features of dumping sites and the surrounding ground features. Based on Landsat images, this study utilized ArcGIS software to visually interpret the extension of the dumping sites. Based on field investigation and comparison of shape, color, texture, etc., on remotely sensed images, unique interpretation marks of the stope and dumping sites were obtained. These were the shape of steps near the stope, different colors based on the shape and grid texture, etc. The vector format boundaries of the dumping sites were visually extracted using the unique interpretation marks.

### 3.2. Calculation of the NDVI

In this study, the *NDVI* was used as the monitoring indicator of vegetation restoration. The *NDVI* can be calculated by the reflectance in the red band and the near-infrared (*NIR*) band [4] according to Equation (1):

(1) $$NDVI=\frac{NIR-R}{NIR+R}$$

where *NIR* is the surface reflectance in the near-infrared band; *R* is the surface reflectance in the red band.

### 3.3. Identification of Vegetation Disturbance and Restoration

#### 3.3.1. Vegetation Disturbance and Restoration Model

**Figure 3:**
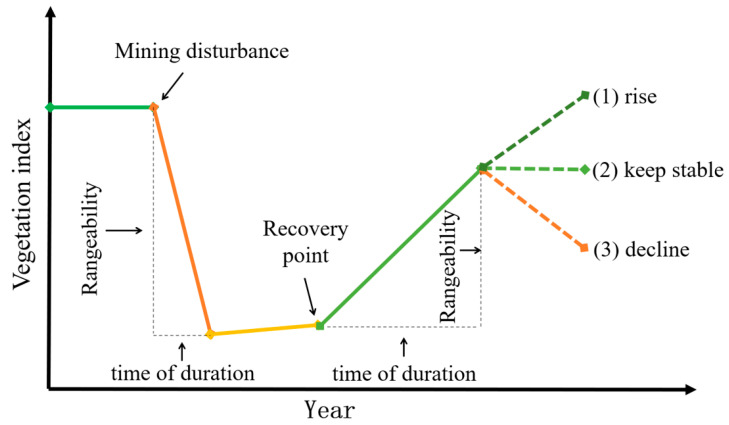
Vegetation disturbance and restoration process of open-pit mines.

For the whole mining process, the NDVI showed different changing characteristics in different mining stages. As shown in Figure 3, the whole mining process could be approximately divided into the following four stages: (1) pre-mining stage; (2) low-intensity mining stage; (3) high-intensity mining stage; (4) ecological restoration stage. In the pre-mining stage, the NDVI was less affected by the mining activities, and its limited change was mainly caused by natural factors such as temperature and precipitation. In the stage of low-intensity mining, the NDVI decreased sharply because the original vegetation coverage began to be damaged. In the stage of high-intensity mining, the mining activities increased, and the damage to the vegetation was continuous. In this time, the NDVI remained stable at the lowest value during the whole process. Due to the damage to vegetation caused by the mining activities, it is not possible to only rely on natural restoration, as this process is slow. Artificial restoration is necessary to enter the ecological restoration stage, during which the NDVI will rise rapidly. However, the NDVI can changes in different ways after restoration, as follows. (1) The NDVI increases. This situation may be due to the continuation of restoration, which progressively improves the vegetation. (2) the NDVI rises to a certain value and remains unchanged. This situation may be due to the end of restoration followed by no further damage and the stabilization of the natural growth. (3) the NDVI increases for a while and then decreases. The reason for this may be that some disturbance occurs after restoration, such as the dumping of gangue, a sharp change in the climate, or the low survival rate of the restored vegetation.

#### 3.3.2. LandTrendr Algorithm

LandTrendr (Landsat-based detection of trends in disaster and recovery) is a method developed for the time series of Landsat images to identify the changes in pixel values with time. In other words, The LandTrendr algorithm allows extracting some key parameters from the NDVI temporal series curve by segments. The algorithm procedure is mainly divided into the following steps.

(1) Extraction of the time series. The image slice sequence is established, and then the time series value of the NDVI is extracted at the pixel scale.
(2) Determination of the vertices of the time series trajectories. Firstly, the parameter “spikeThreshold” is set to control the removal of some NDVI outliers caused by residual clouds, shadows, and other noise. Secondly, potential vertices are identified based on the residual criterion and angle threshold method.
(3) Fitting of time series trajectories. Two-time series trajectories are constructed based on point-to-point connection and regression fitting. By comparing the mean-square error (MSE), the connection method with a small MSE is selected as the time series trajectory.
(4) Trajectory simplification and optimal trajectory selection. The iterative method is used to simplify and redefine the trajectory. The trajectory with the number of segments from “maxsegments” to 1 is generated, and then the *p*-value of *F*-statistics is used to select the optimal trajectory.

LandTrendr provides many parameters to control the extracted model information, which can be used to monitor the change of vegetation in the whole process of mining [35]. The input and output parameters of the LandTrendr algorithm are shown in Table 2. In this paper we adopted default values for the parameters.

#### 3.3.3. Temporal and Spatial Characteristics of Vegetation Disturbance and Restoration

The temporal characteristics of vegetation disturbance and restoration mainly include the initial time and duration. The initial time refers to the year when the vegetation index obviously changed for the first time due to disturbance or restoration events. The NDVI in the initial year of vegetation disturbance usually decreases sharply when mining activities or land occupation occur. When artificial restoration is involved, the NDVI in the initial year of vegetation restoration usually increases sharply. The vegetation changing characteristics in the study area can also be reflected by the duration of vegetation disturbance and restoration. The spatial characteristics of vegetation disturbance and restoration mainly refer to the intensity of vegetation disturbance and restoration, namely, the changing range of the NDVI.

Based on the vegetation disturbance and restoration model and LandTrendr algorithm, this study calculated the NDVI changes in the Baorixile open-pit mining area for a total of 32 years from 1990 to 2021. In order to avoid the impact of the starting point error, the two years of 1990 and 2021 were excluded. Therefore, the NDVI changes from 1991 to 2020 were monitored and identified, and the initial time and duration of vegetation disturbance and restoration in the mining area were obtained.

## 4. Results and Discussion

### 4.1. Analysis of Temporal and Spatial Changes of the Boundaries of the Dumping Sites

**Figure 4:**
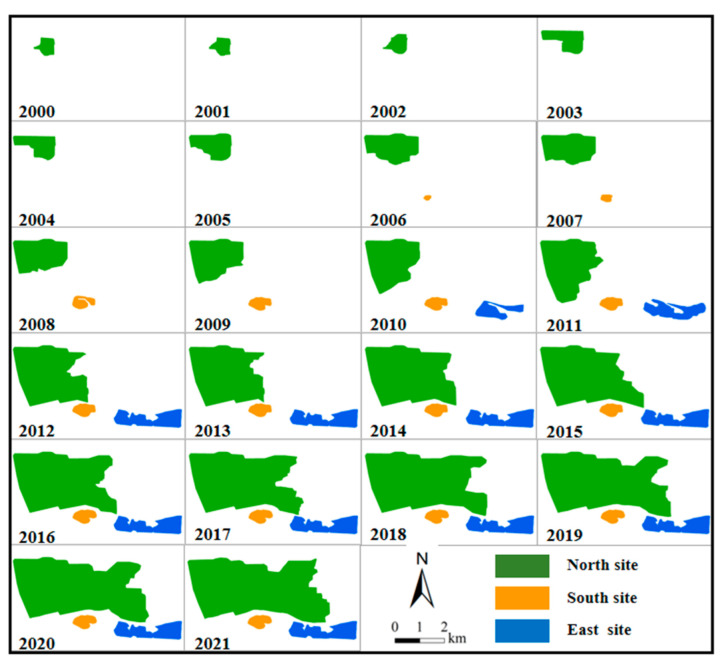
Results of the boundary extraction for the dumping sites from 2000 to 2021.

**Figure 5:**
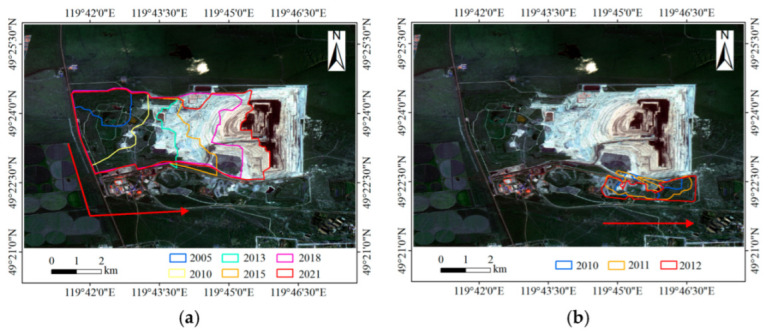

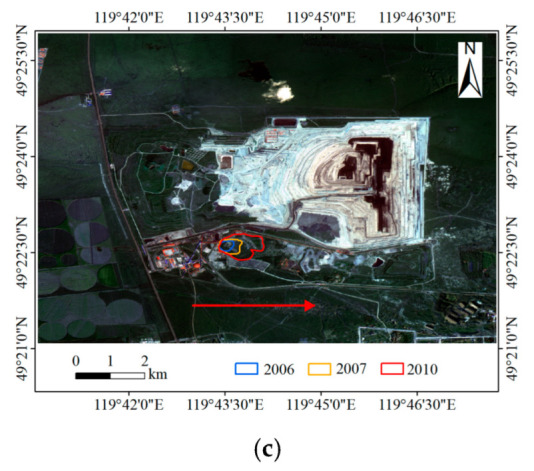
Expansion directions (red arrows). (**a**) North site; (**b**) East site; (**c**) South site.

Based on the remotely sensed images, it was found that the first dumping site appeared in 2000. Therefore, this section used the Landsat images from 2000 to 2021 to visually identify the boundaries of the dumping sites. The results are shown in Figure 4, and the expansion directions of the dumping sites are shown in Figure 5.

The mining area adopted the mining mode of “mining while filling”. Since the appearance of the North site in 2000, its area increased year by year. In terms of expansion direction, the North site first expanded to the southeast along the mining area boundary and began to expand to the east after 2011. In 2006, the South site began to appear. From 2006 to 2009, the South site began to be used, and its area increased. In 2010, the South site stopped being used, and the East site appeared. In the next few years, the east part of the North site and the East site served as the main waste disposal sites, and the area of these two sites increased. The East site stopped being used in 2012. While the areas of the South site and East site increased, there was almost no significant change in their expansion direction; the South site expanded slightly to the East.

**Figure 6:**
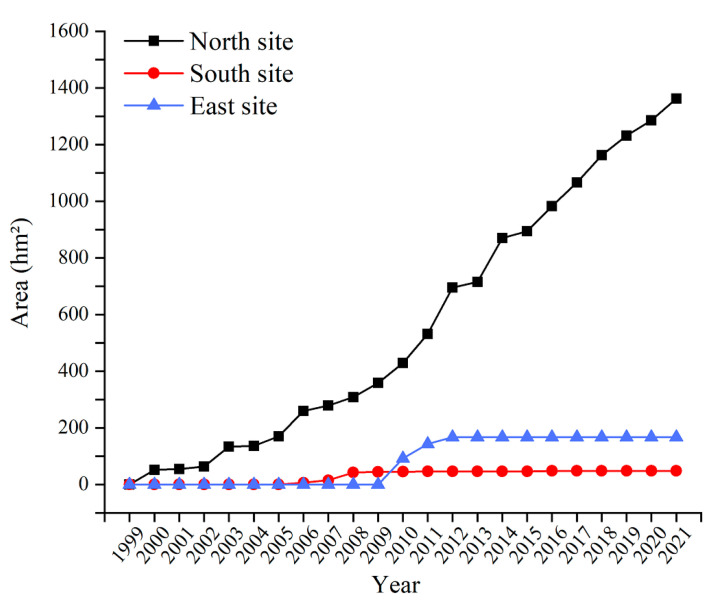
Areas of North site, South site, and East site.

In order to further analyze the changes in each dumping site, this paper performed statistical analyses on the area of each dumping site from 2000 to 2021, and the results are shown in Figure 6. In 1999, the area occupied by dumping sites in the Baorixile open-pit mine was zero. The overall area of the South site was small, and gradually expanded from 2006 to 2010, increasing from 6.47 hm^2^ in 2006 to 45.26 hm^2^ in 2010. It basically remained the same at about 45.96 hm^2^ from 2010 to 2021. The area of the East site increased rapidly from 2010 to 2012, remained basically unchanged from 2013 to 2021, and then stabilized at about 167 hm^2^. Since the appearance of the North site in 2000, its area increased greatly, reaching 1362.16 hm^2^ in 2021. It was used as the main dumping site.

### 4.2. Results of the NDVI

**Figure 7:**
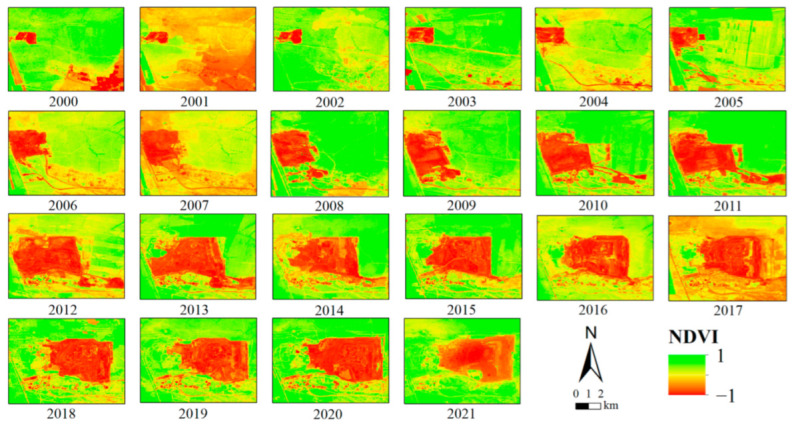
NDVI variation from 2000 to 2021.

Figure 7 shows the temporal and spatial distribution of the NDVI in the study area from 2000 to 2021. In Figure 7 the location of stope is indicated in red, and there the NDVI presents the lowest value. The NDVI around the stope is lower than in the far area. The color of the three dumping sites in the Figure change from red to green, and the NDVI gradually increased.

### 4.3. Monitoring of Vegetation Restoration at the Dumping Sites

#### 4.3.1. Temporal Characteristics of Vegetation Disturbance and Restoration

(1) Analysis of initial time of vegetation disturbance and restoration

**Figure 8:**
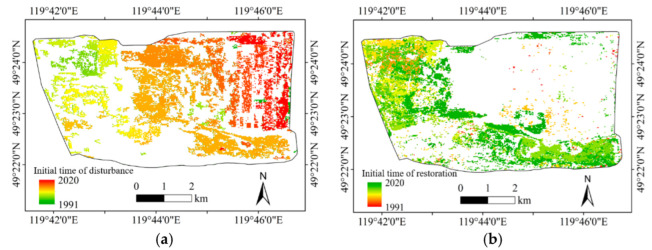
Initial time of vegetation disturbance and restoration. (**a**) Initial time of disturbance; (**b**) Initial time of restoration.

The initial time of vegetation disturbance and restoration acquired by LandTrendr are shown in Figure 8. It can be seen that with the passage of time, the vegetation disturbance first moved to the southeast and then to the East. Vegetation restoration began around 2000, mainly in the northwest of the North site and then in the East site and the South site.

**Figure 9:**
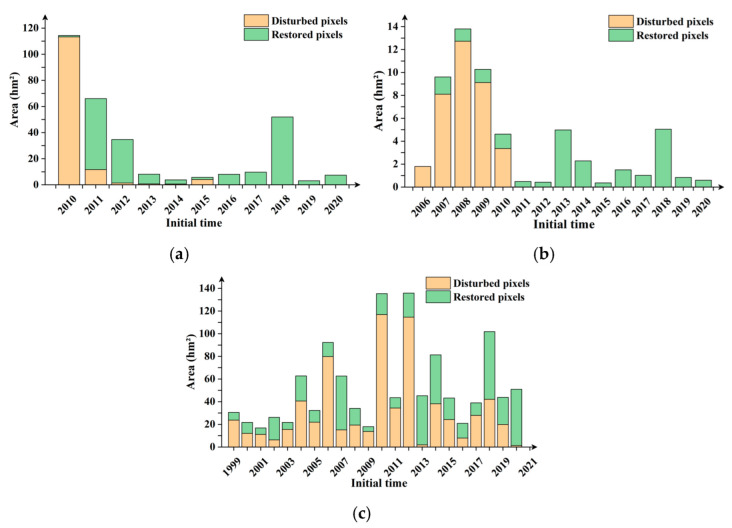
Pixel statistics of disturbance and restoration initial time. (**a**) East site; (**b**) South site; (**c**) North site.

In order to further observe the initial time characteristics of disturbance and restoration, the disturbance and restoration areas of the East site from 2010 to 2020, the South site from 2006 to 2020, and the North site from 1999 to 2020 were examined. The statistical results are shown in Figure 9.

It can be seen from the statistics results that the East site began to dump and accumulate waste in 2010, resulting in vegetation disturbance. The main disturbance time occurred in 2010 and 2011, and the restoration began in 2011, with a significant effect in 2011 and 2018. Vegetation disturbance occurred in the South site in 2006. The main disturbance occurred from 2007 to 2009, and the restoration began simultaneously in 2007. The North site accumulated waste since 1999, and the vegetation restoration started at the same time, increasing in the following years.

(2) Analysis of the duration of vegetation disturbance and restoration

**Figure 10:**
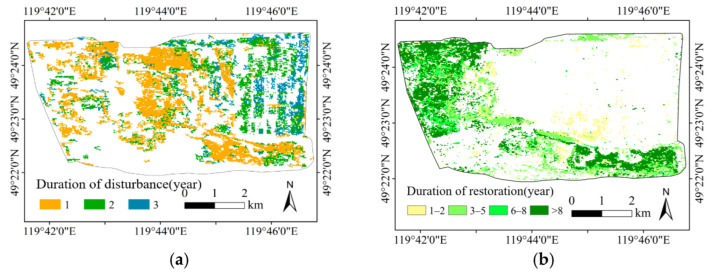
Duration of vegetation disturbance and restoration. (**a**) Duration of disturbance; (**b**) Duration of restoration.

From the curve of mining vegetation disturbance and restoration model in the mining area, it can be seen that mining, land occupation, accumulation of dumping sites, and artificial restoration occurred as a continuous process. It is generally believed that the persistence of a low vegetation index indicates the persistence of vegetation disturbance caused by mining, and the persistence of a high vegetation index indicates the persistence of artificial restoration. The monitoring results of vegetation disturbance and restoration duration are shown in Figure 10.

Figure 10 shows that the vegetation disturbance was mainly concentrated in the stope and each dumping site, while restoration was mainly concentrated at each dumping site. The duration of vegetation disturbance was short, but that of the restoration was generally long. In order to further analyze the duration of vegetation disturbance and restoration at each dumping site, the number and area of the pixels corresponding to the duration of these processes at each dumping site were determined. The results are shown in Table 3 and Table 4.

It can be seen from the statistics results that the duration of vegetation disturbance caused by mining was mainly concentrated in 1–3 years, with most pixels present for 1 year and few pixels present for 3 years. The time of vegetation restoration was generally long, and the pixels with a duration greater than 8 years accounted for a large proportion.

The characteristics of the duration of vegetation disturbance and restoration of the Baorixile open-pit mining area re clearly reflected by the results. The mining disturbance of the Baorixile open-pit mining area was relatively sharp, and the restoration occurred after a short time from the start of the mining disturbance. The duration of vegetation restoration of the whole open-pit mining area was long and evenly distributed. The South site and the East site were filled in a short time, and then repair events started. In the early stage of the mining activities, a certain vegetation restoration was carried out in the North site. This may be related to the behavior of mining enterprises to carry out reclamation while mining coal, so to realize the integration of mining and reclamation.

#### 4.3.2. Spatial Characteristics of Vegetation Disturbance and Restoration

**Figure 11:**
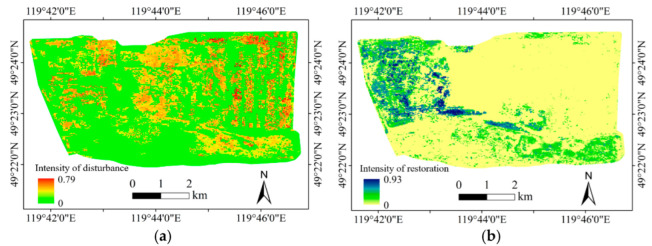
Intensity of disturbance and restoration of the vegetation. (**a**) Intensity of the disturbance; (**b**) Intensity of restoration.

The spatial distribution of the intensity of vegetation disturbance and restoration were identified, and the results are shown in Figure 11.

The vegetation disturbance area was evenly distributed in the whole open-pit mining area, mainly in the stope and each dumping site (Figure 11). The vegetation disturbance in the stope was caused by land stripping, and the vegetation disturbance in the dumping sites was caused by land occupation. The vegetation restoration area was mainly concentrated in the South site, East site, and the northwest of the North site. In the northwest of the North site, the restoration effect was obvious; furthermore, the East site and the South site were also well restored.

In our study area, it is the mining activity that caused the disturbance; so we associated disturbance with restoration while evaluating the effect of vegetation restoration. In order to further analyze the effect of vegetation restoration, this study defined the average intensity of disturbance and restoration of the dumping sites. The average disturbance intensity was defined as the sum of the disturbance intensity of all disturbed pixels divided by the total number of disturbed pixels (Equation (2)). The average restoration intensity was defined as the sum of the restoration intensity of all restored pixels divided by the total number of restored pixels (Equation (3)). The average vegetation disturbance intensity and average vegetation restoration intensity of the three dumping sites were determined. The results are shown in Table 5. It can be seen from the statistics results that the average restoration intensity of each dumping site was greater than the average disturbance intensity. The disturbed areas were well restored, and the measure of “mining while repairing” may be the reason. In a short time after the disturbance, the dumping sites were restored artificially to improve the environment of the mining area.

(2) $$AI_{dis}=\frac{S_{dip}}{T_{dp}}$$

(3) $$AI_{res}=\frac{S_{rip}}{T_{rp}}$$

In the equations, *AI_dis_* represents the average disturbance intensity, *S_dip_* represents the sum of the pixels indicating the disturbance intensity, *T_dp_* represents the total number of disturbed pixels, *AI_res_* represents the average restoration intensity, *S_rip_* represents the sum of the pixels indicating the restoration intensity, and *T_rp_* represents the total number of restoration pixels.

## 5. Conclusions

This paper examined the Baorixile open-pit mine in Hulunbuir and used Landsat images taken in 32 years, from 1990 to 2021, to analyze the temporal and spatial changes of vegetation disturbance and restoration based on the NDVI and LandTrendr algorithm. Some conclusions were reached as follows:

(1) By analyzing the vegetation disturbance and restoration from a temporal perspective, we found that the vegetation disturbance in the dumping sites was relatively rapid, and the restoration started in a short time (0~2 years) after land occupation. The duration of vegetation disturbance was mainly 1–3 years, with most pixels lasting for 1 year, and fewest pixels lasting for 3 years. The duration of vegetation restoration was generally long, lasting over 8 years for the great majority of pixels.
(2) By analyzing the vegetation disturbance and restoration from a spatial perspective, we found that vegetation restoration was mainly concentrated in the South site, the East site, and the northwest of the North site. The average restoration intensity of each dumping site was greater than the average disturbance intensity.
(3) This study demonstrates that temporal remote sensing images can clearly trace the vegetation restoration process of an open-pit mine. Combined with the LandTrendr algorithm, these images can be used to monitor the progress and quality of ecological restoration projects such as vegetation restoration in mining areas.

In the future, more open-pit coal mines will be investigated to reveal the general spatio-temporal patterns of vegetation disturbance and restoration, which will provide more support for the planning of ecological remediation.

## Figures and Tables

**Table 1:** Band information of the images used for calculating the NDVI.

Satellite	Band Number	Band Name	Wavelength (μm)	Spatial Resolution (m)
Landsat 5	Band 3	Red	0.63–0.69	30
	Band 4	Near-infrared	0.76–0.90	30
Landsat 7	Band 3	Red	0.63–0.69	30
	Band 4	Near-infrared	0.76–0.90	30
Landsat 8	Band 4	Red	0.64–0.67	30
	Band 5	Near-infrared	0.85–0.88	30

**Table 2:** LandTrendr parameters (Quote from LT-GEE Guide).

Parameter	Definition	Default
maxSegments	Maximum number of segments fitted on a time series.	-
spikeThreshold	Threshold for dampening the vertices of the time series (1.0 means no dampening).	0.9
vertexCountOvershoot	Initial regression-based monitoring of potential vertices can make vertices more than (maxSegments + 1). The value may be outside this range. If exceeded, angle-based culling can be used to return to the desired number of vertices. Allows a mixture of methods for vertex recognition.	3
preventOneYearRecovery	Prevent recovery events that take only one year to complete.	False
recoveryThreshold	If the recovery rate of the recovery segment in the time series is higher than 1/recoveryThreshold (in years), the segment is not allowed, and segments with different thresholds must be used.	0.25
pvalThreshold	If the *p*-value of the fitting model exceeds this threshold, the current model should be discarded, and the Levenberg-Marquardt optimizer should be used to fit another model.	0.1
bestModelProportion	When selecting the optimal model, the difference between the *p*-value model with the most vertices and the model with the least vertices is at most this proportion.	1.25
minObservationsNeeded	The minimum observations required to output the fitted values.	6
TimeSeries	Collection of remote sensing images used to extract trajectory trends.	1990–2021

**Table 3:** Duration of the disturbance.

	Duration of Disturbance (Years)	Number of Pixels	Area (hm^2^)
North site	1	6007	360.42
	2	2171	130.26
	3	721	43.26
South site	1	295	17.7
	2	135	8.1
	3	3	0.18
East site	1	1660	99.6
	2	476	28.56
	3	76	4.56

**Table 4:** Duration of restoration.

	Duration of Restoration (Year)	Number of Pixels	Area (hm^2^)
North site	1–2	1518	91.08
	3–5	1554	93.24
	6–8	1187	71.22
	>8	3678	220.68
South site	1–2	16	0.96
	3–5	122	7.32
	6–8	83	4.98
	>8	188	11.28
East site	1–2	306	18.36
	3–5	740	44.4
	6–8	190	11.4
	>8	1548	92.88

**Table 5:** Average intensity of disturbance and restoration of the vegetation.

	Average Intensity of Disturbance	Average Intensity of Restoration
North site	0.371	0.515
East site	0.320	0.415
South site	0.398	0.489

## Data Availability

The Landsat data can be downloaded from GEE (https://developers.google.com/earth-engine/datasets/, accessed on 15 December 2021).
